# Space Optimized Plane Wave Imaging for Fast Ultrasonic Inspection with Small Active Aperture: Simulation and Experiment

**DOI:** 10.3390/s21010055

**Published:** 2020-12-24

**Authors:** Hao Sui, Pan Xu, Jinxing Huang, Hongna Zhu

**Affiliations:** 1 School of Physical Science and Technology, Southwest Jiaotong University, Chengdu 610031, China; suihaoabc@163.com; 2 School of Meteorology and Ocean, National University of Defense Technology, Changsha 410073, China; xupan09@nudt.edu.cn (P.X.); bbg1528@163.com (J.H.)

**Keywords:** ultrasonic arrays, defect characterization, ultrasonic imaging, plane wave imaging, total focusing method

## Abstract

Plane wave imaging (PWI) is attracting more attention in industrial nondestructive testing and evaluation (NDT&E). To further improve imaging quality and reduce reconstruction time in ultrasonic imaging with a limited active aperture, an optimized PWI algorithm was proposed for rapid ultrasonic inspection, with the comparison of the total focusing method (TFM). The effective area of plane waves and the space weighting factor were defined in order to balance the amplitude of the imaging area. Experiments were carried out to contrast the image quality, with great agreement to the simulation results. Compared with TFM imaging, the space-optimized PWI algorithm demonstrated a wider dynamic detection range and a higher defects amplitude, where the maximum defect amplitude attenuation declined by 6.7 dB and average attenuation on 12 defects decreased by 3.1 dB. In addition, the effects of plane wave numbers on attenuation and reconstruction time were focused on, achieving more than 10 times reduction of reconstruction times over TFM.

## 1. Introduction

Ultrasonic phased arrays have attracted more and more attention in nondestructive testing and evaluation (NDT&E), due to its ability for achieving high-sensitivity dynamic focus scanning in a wide range without moving the transducer, compared with conventional ultrasonic testing [1,2,3,4,5,6]. At present, phased array imaging is usually formed using the synthetic aperture focusing technique (SAFT) or total focusing method (TFM) [7,8,9]. In particular, TFM is based on the full matrix capture (FMC), which contains every emitting–receiving combination in post-processing imaging. Because it provides the best resolution and a larger dynamic range, TFM is usually considered the “gold standard” in the NDT&E field [10,11].

However, TFM requires more computing time to deal with the FMC matrix. For a TFM imaging process, the costed time is determined by the size of the imaging area, imaging resolution, and the number of array elements. Some methods are adopted to reduce calculations, such as making use of sparse matrix, using multiple array elements to transmit chirp signals instead of ordinary pulse excitation signals, and achieving data processing with the GPU [12,13,14,15]. Whereas the quality of the TFM image is positively related to the amount of its post-processing data matrix, reducing the data amount used in the imaging processing worsens the quality of the TFM image. Thus, a method with less computation time is extremely desirable for real-time monitoring and inspection in practice.

As a very promising method to increase the frame rate, plane wave imaging (PWI) has attracted extensive research interests recently [16,17]. In PWI, a specific time delay is set to emit plane waves at different angles in the medium, and PWI imaging at a certain angle is formed by delay-and-sum methods in all array elements. Thus, the images at all angles are coherently superimposed to form a PWI imaging. Previously, the PWI method has been applied in industry and realized an equal or higher image quality, with obvious reduction of computation time compared to the TFM method. The plane wave with the phase coherence imaging technique was proposed, which provided similar or superior image quality than TFM at significantly higher frame rates [18]. The PWI of cracks in the Fourier domain was realized by reducing the computation times by a factor up to 13 [19]. The quality of TFM imaging and PWI imaging with a 128 elements array was tested, and three to ten times less transmissions than with the TFM method were achieved [20].

However, all previous works of PWI imaging were based on a 64 or 128 elements linear array. In NDT&E inspection, the element number in a typical industrial array is usually less than 64 due to the limitation of the test specimen with complex shapes and the difficulty of coupling. Indeed, the imaging quality of PWI in a limited active aperture is desired in industrial applications. In addition, the plane wave emitted by linear array is highly directional, which leads to the lopsidedness of the amplitude distribution on the final PWI image and the reduction of the imaging quality. Therefore, larger detection range with lower attenuation on defect amplitude should be investigated using new methods.

Here, we developed an algorithm to optimize PWI by weighting the imaging area spatially. A space weighting factor was presented to equalize the amplitude and distribution of the PWI images. As a typical and widely used industrial probe, the 32-element linear array was applied to analyze the imaging quality and reconstruction time of the proposed method. Meanwhile, the results of TFM imaging under the same conditions were compared. The effect of the plane wave numbers utilized on the imaging result was discussed in detail, resulting in the least imaging time with similar imaging quality. The remainder of this paper is organized as follows. In Section 2, the theory of the TFM and PWI methods are analyzed, and the proposed space weighting factor is focused on. The simulation and experimental results obtained with the PWI and TFM algorithms were investigated; additionally, the comparison of PWI imaging under different angle parameters is displayed in Section 3. Conclusions are given in Section 4.

## 2. Theory and Model

### 2.1. Optimized PWI Algorithm

Superimposed by a set of *M* plane waves, the PWI image included the *M* times array emitting process. Thereinto, the plane wave was generated by the array elements according to a specific delay, then the echo signals were recorded on each element.

**Figure 1:**
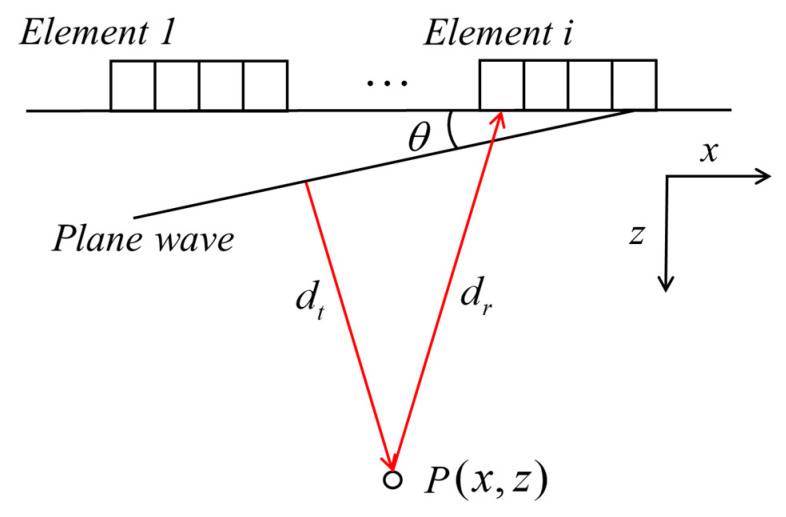
Reception at element *i* while *θ* plane wave emitting.

The schematic diagram of reception at element *i* while θ plane wave emitting is shown in Figure 1. The *x-z* plane was the target area to be imaged. The number of array elements was *N*, and plane waves of *M* angles were emitted. The coordinates of each array element are listed as $\left(x_{i},z_{i}\right)$. To excite a plane wave with θ angle, the delay time applied to each element was:

(1) $$\tau \left(\theta ,x_{i}\right)=\frac{x_{i}\cdot \sin\theta }{c}$$

where *c* is the velocity of ultrasonic waves. For point P(x,z) in the imaging area, the propagated distance of plane waves from the array to P(x,z) is $d_{t}$, and the propagated distance of the back-scattered signal from P(x,z) to the *i*th element is set to $d_{r}$. Thus, the propagation time corresponding to this process is:

(2) $$\tau \left(\theta ,x_{i},x,z\right)=\frac{x\sin\theta +z\cos\theta +\sqrt{z^{2}+{\left(x_{i}-x\right)}^{2}}}{c}$$

The amplitude of each pixel was obtained by the coherent superposition of *M* emission angles and each receiving array element, which can be written as:

(3) $$A_{\mathrm{PWI}}\left(x,z\right)=\overset{N}{\underset{i=1}{\sum }}\overset{M}{\underset{j=1}{\sum }}F_{ij}\left(\tau \left(\theta ,x_{i},x,z\right)\right)$$

where $F_{ij}$ is the ultrasonic signal received by the *i*th array elements at the *j*th angled plane wave emitted.

**Figure 2:**
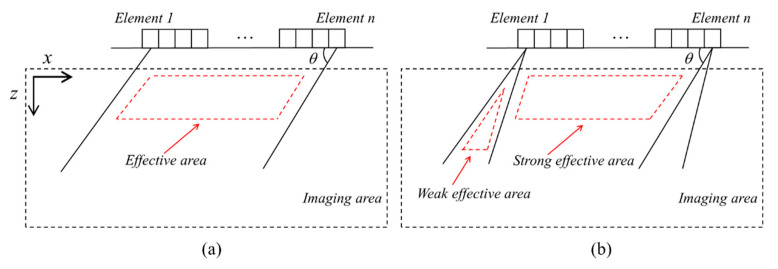
The effective area of plane wave imaging (PWI) at (**a**) θ angle (**b**) multi-plane wave mode.

The effective area of plane waves should be mentioned. The emitted plane wave at a specific angle can be considered to propagate in a limited area in the imaging area due to its strong directivity of ultrasonic energy, as seen in Figure 2a. The effective imaging region $E\left(x_{1},x_{n},z,\theta \right)$ at *z* depth is related to the active aperture *L* and the angle of the plane wave θ [20]:

(4) $$E\left(x_{1},x_{n},z,\theta \right)\in \left[x_{1}+\frac{z}{\tan\theta },x_{n}+\frac{z}{\tan\theta }\right]$$

where $x_{1}$ and $x_{n}$ are the *x*-coordinates of the first and last array element, and *z* is the imaging depth. Figure 2b represents the case of multi-plane wave imaging, the central area under the array reached by plane waves with most angles, which is named as a strong effective area in our work. The reduction in the number of plane waves indicates a cut-down of PWI image intensity, which is defined as a weak effective area, as illustrated in Figure 2b.

Usually, uneven amplitude distribution in the imaging area causes the active detection range to diminish, leading to poor imaging amplitude and signal-noise ratio (SNR) for zones far away from the array. In industry fields, the effective aperture of the array is generally small. Thus, the space weighting factor ω(x,z) is produced to equalize the intensity of the imaging area, for achieving a larger dynamic monitoring range with a limited active aperture. Here, the ω(x,z) is defined as:

(5) $$\omega \left(x,z\right)=\overset{M}{\underset{j=1}{\sum }}s_{j}\left(x,z,\theta \right)$$

where $s_{j}\left(x,z,\theta \right)$ is the amplitude distribution function with a θ angle. In case of the pixel point belonging to the effective imaging area, the $s_{j}\left(x,z,\theta \right)$ is set to 1, otherwise, it is 0, which is expressed as:

(6) $$s_{j}\left(x,z,\theta \right)=\{\begin{array}{c} 0,\;if\left(x,z\right)\notin E\left(x_{1},x_{n},z,\theta \right)\\ 1,\;if\left(x,z\right)\in E\left(x_{1},x_{n},z,\theta \right) \end{array}$$

In the strong effective area, ω(x,z) is much higher than that of the weak effective area. The optimized PWI imaging $I_{\mathrm{PWI}}\left(x,z\right)$ under a specific emitting-reception process is the ratio of the initial amplitude of the PWI image to the space weighting factor ω(x,z), for the achievement of equalizing the intensity of the imaging area:

(7) $$I_{\mathrm{PWI}}\left(x,z\right)=\frac{A_{\mathrm{PWI}}\left(x,z\right)}{\omega \left(x,z\right)}$$

### 2.2. TFM Algorithm

In the TFM process, array elements are sequentially induced, and ultrasonic signals are received by all array elements simultaneously. At the same time, TFM imaging is performed by considering the times-of-flights from all emitting and receiving processes to the imaging area, achieving a focus on emission and reception.

**Figure 3:**
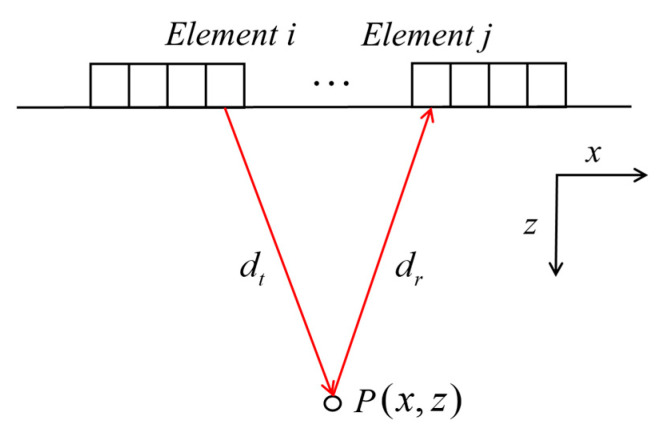
Reception at array element *i* while element *j* emitting.

The principle of TFM is shown in Figure 3. In the two-dimensional coordinate system, the coordinate *x* axis is the scanning direction of the phased array. The number of phased array elements is *N*. The propagation time, when the ultrasonic wave is transmitted from the element *i* through P(x,z) to the received element *j*, is obtained from:

(8) $$\tau \left(x_{i},z_{i},x_{j},z_{j}\right)=\frac{\sqrt{{\left(x_{i}-x\right)}^{2}+{\left(z_{i}-z\right)}^{2}}+\sqrt{{\left(x_{j}-x\right)}^{2}+{\left(z_{j}-z\right)}^{2}}}{c}$$

The value of each pixel $I_{TFM}\left(x,z\right)$ is calculated by the superposition of corresponding amplitude achieved from each transmitting-receiving signal, which is written as:

(9) $$I_{\mathrm{TFM}}\left(x,z\right)=\overset{N}{\underset{i=1}{\sum }}\overset{N}{\underset{j=1}{\sum }}F_{ij}\left(\tau \left(x_{i},z_{i},x_{j},z_{j}\right)\right)$$

## 3. Results and Discussion

### 3.1. Simulation and Experiment Setup

**Figure 4:**
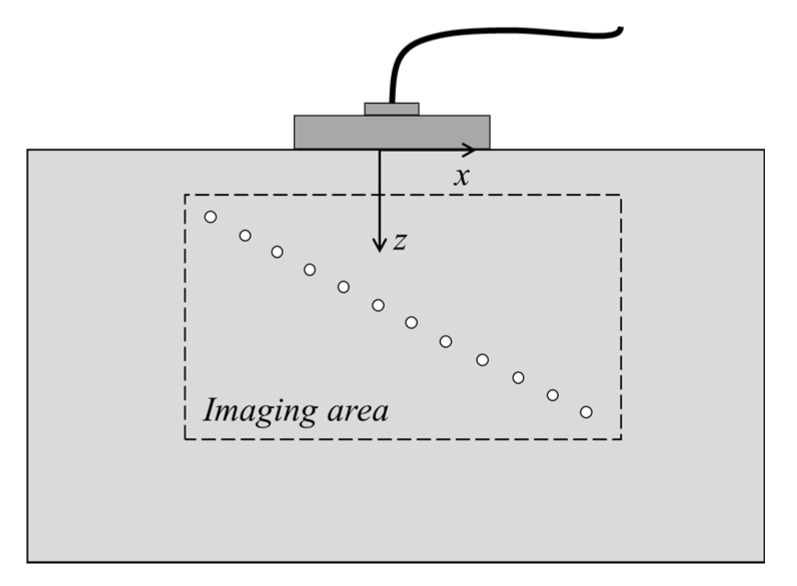
The testing specimen and defects in the simulation and experiment.

A series of simulations and experiments were performed to verify the operation of the proposed PWI fast imaging method. Simulations were based on CIVA, which is a well-known sound field and defect response simulation software. The schematic representation of the defects and inspection setup is exhibited in Figure 4. The specimen was plain steel with a size of 300 mm × 150 mm. Additionally, the specimen contained twelve 2 mm diameter side-drilled holes at different depths. After calculation, the longitudinal wave velocity in the tested specimen was 5930 m·s^−1^. A 32-element linear array was set on the top surface of the tested specimen to generate plane waves and receive the ultrasonic signals. Plane waves were emitted by setting corresponding delay laws to array, then the echo signals were received by the array with null delay laws. In the same way, the emitting and receiving array were placed in the same geometric location. Array parameters are listed in Table 1.

Meanwhile, the experimental setup was established, where the parameters of array and defects were the same as those used in the simulation. The PWI acquisition process included the emission of 61 plane waves with different angles and reception at every element. The angle of the plane waves ranged from −60° to 60°, with a scanning step of 2°. We used a Multi2000 manufactured by M2M and a linear array 5L32-A11 from Olympus to carry out data collection processing. Thus, a 61×32 PWI data matrix and 32×32 FMC matrix were obtained numerically and experimentally.

### 3.2. Comparison of PWI and TFM Imaging

Next, the imaging quality of the two aforementioned algorithms was demonstrated based on the previous simulation and experiment setup. In the same imaging area, TFM and PWI imaging were formed using the 32×32 FMC data matrix and 61×32 PWI data matrix, respectively. Considering that one element was activated at one emission in TFM, while the whole array elements were excited simultaneously in PWI, hence, the amplitude of plane waves was much higher than cylindrical waves generated in TFM. In the experiment, the gain difference between PWI and TFM was fixed to 20 dB in order to reach an equal amplitude of the defect echoes.

**Figure 5:**
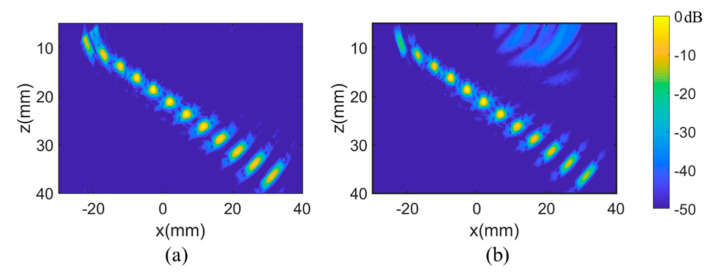
Simulated images obtained by the (**a**) PWI and (**b**) total focusing method (TFM) algorithms.

The simulation images obtained by PWI and TFM are displayed in Figure 5, with similar results obtained by the two methods. The level of amplitude of images is usually expressed with the decibel units, which is defined as:

(10) $$I_{dB}\left(x,z\right)=20{log}_{10}\frac{I\left(x,z\right)}{I_{max}}$$

where I(x,z) is the amplitude calculated in Equations (6) and (8). $I_{max}$ is a certain value in each imaging, corresponding to the maximum amplitude in the imaging area.

The noise that appears in the upper side of Figure 5b was less than −35 dB, which was caused by the delay-and-sum algorithm used in the TFM method. In this simulation, the times-of-flights of the noised area were close to the echo signals caused by the shallow defects; thus, there are circular noises in the upper right of Figure 5b. In regard to the two methods, the defects in the middle of Figure 5 are at similar levels, but at the position farthest from the array (i.e., in the top and bottom of the imaging area), the PWI provides a lower amplitude attenuation than that of the TFM, which indicates the PWI owing a higher detection range and capability.

**Figure 6:**
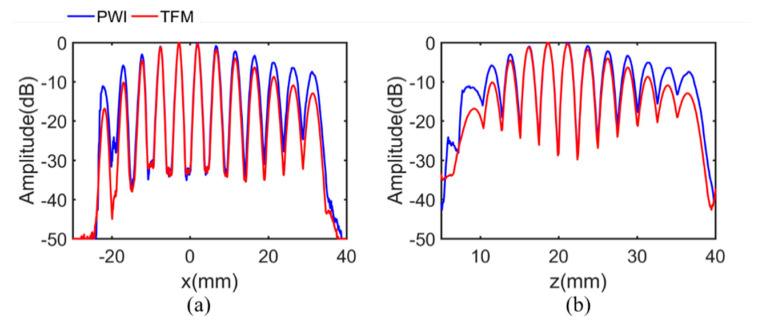
The projection of the maximum amplitude along the (**a**) *x* direction. The (**b**) *z* direction in simulation.

Figure 6 illustrates the echo dynamic curve in *x* direction and *z* direction, which are defined as the maximum of the projection along the *x* and *z* axis. It can be seen from Figure 6 that there is a larger dynamic detection range in the PWI image, further showing the advantages of PWI. Compared with the defects just below the array, the amplitude attenuation of the defects away from the array is significantly declined. For the sake of completeness, the attenuation of each diameter hole by the two methods is listed in Table 2. Defects are numbered according to depths in the *z* direction. In the PWI method, the average attenuation on 12 defects was reduced by 2.6 dB, and the maximum attenuation decreased by 5.8 dB, compared with TFM.

**Figure 7:**
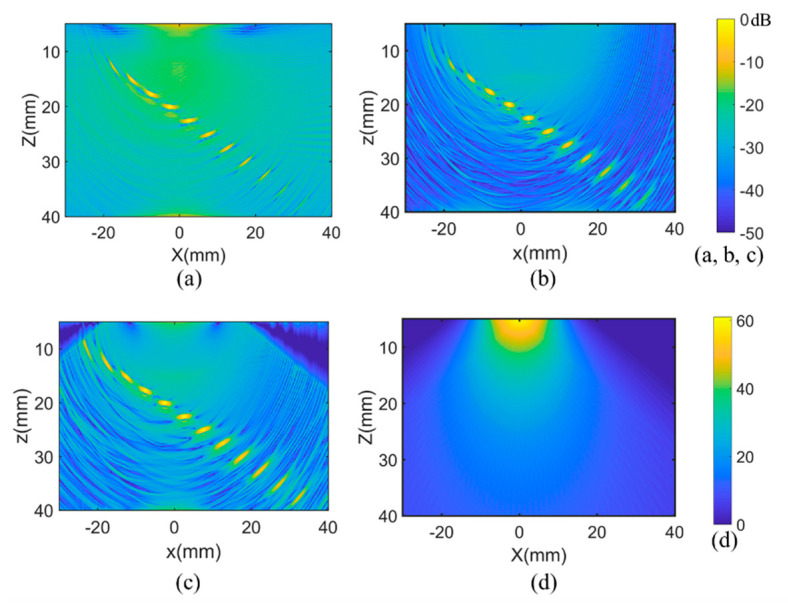
Experimental images obtained by the (**a**) conventional PWI, (**b**) TFM algorithms, and (**c**) optimized PWI with (**d**) space weighting factor.

The experimental images of conventional and optimized PWI and TFM cases are demonstrated in Figure 7, where the experimental results are in good consistency with the simulation. In the conventional PWI results shown in Figure 7a, compared with the optimized PWI and TFM, the background noise is relatively higher and the amplitude of the defects far away from the array are much lower. This indicates that the detection range of the conventional PWI method is narrower than the optimized PWI and TFM, and only the defects below the array are well presented. The result of optimized PWI is shown in Figure 7b; due to the limitation of the angle scanning range (i.e., from −60° to 60°) set in the data collection progress, the upper left and upper right corners of the image are not involved in the effective imaging area. Thus, the amplitude value is less than −40 dB. The space weighting factor in the imaging area is illustrated in Figure 7d, where the amplitude of weight decreases as the distance away from the array increases. The defects right below the array weigh around 30; however, the weights of defects in remote locations are dozens, which leads to the larger defect amplitude decreasing and bad imaging quality for the remote defects in the conventional method.

**Figure 8:**
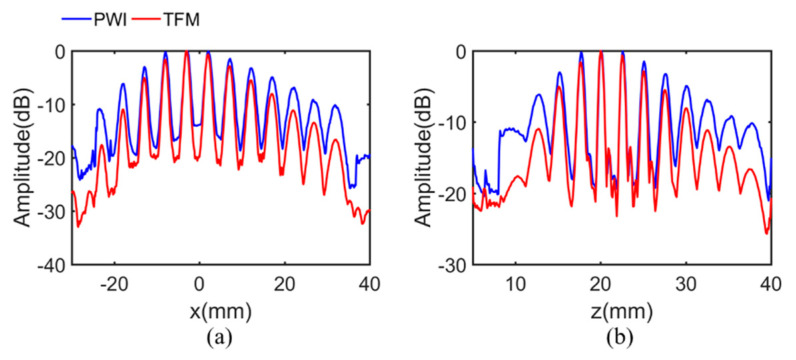
The projection of the maximum amplitude along the (**a**) *x* direction. The (**b**) *z* direction in the experiment.

The background noise of the optimized PWI and TFM was about −30 dB, which is mainly manifested in artifacts caused by the algorithm. The noise of the PWI image was slightly greater than that of the TFM, which was lower than 3 dB on most pixels in the imaging area. At the same time, all defects are well presented in the two images. Note that all the amplitude attenuations of defects in the PWI image were lower than those of TFM, whether the defects were located far away from the array or not. This is consistent with the simulation. Furthermore, the echo dynamic curves of the *x* and *z* directions are shown in Figure 8. Amplitude attenuations of defects located away from array display an obviously decline, demonstrating that there is a larger active detection range and higher defects amplitude in PWI imaging. In detail, the amplitude attenuation of each defect in the experiment is shown in Table 3. It can be seen more clearly that the amplitude attenuation of PWI among all defects is lower than that of TFM. The maximum attenuation declined by 6.7 dB with the average attenuation being reduced by 3.1 dB.

In addition to the capability in defect detection, the computation time is another important factor of the algorithm. In the experiment, the data acquisition time of the two methods was equivalent, with both times both being less than 1 s. In this case, only the reconstruction time of the image is important, with the data acquisition time on hardware being ignored. In this work, MATLAB 2017a was used as the data post-processing software with an Intel 9900 k CPU. The reconstruction time by two algorithms in the simulation and experiment is listed in Table 4. The costed time of the PWI image formed by 61 plane waves was reduced by about two-thirds compared with the 32-element TFM imaging.

### 3.3. Effects of PWI Parameters on Attenuation and Reconstruction Time

**Figure 9:**
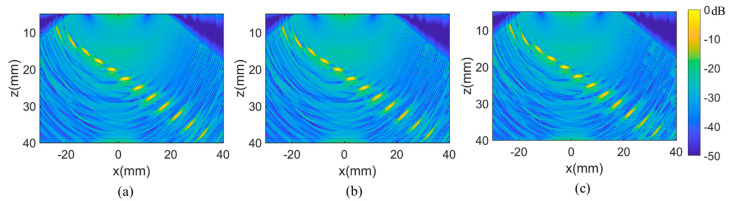
The optimized PWI imaging of (**a**) 61, (**b**) 31, (**c**) and 16 plane waves.

**Figure 10:**
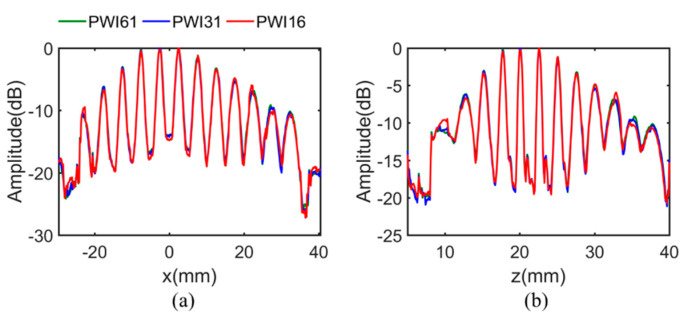
The projection of the maximum amplitude along the (**a**) *x* direction. The (**b**) *z* direction under the case of different plane waves number.

It should be mentioned that higher calculation efficiency is achieved by reducing the angle number in the superposition of single frame image. Hence, the number of plane waves employed in PWI imaging was further analyzed, both in the capability of defect detection and reconstruction time. The space optimized PWI images formed by the superposition of plane waves at 61, 31, and 16 angles are explored in Figure 9. All the scans range from −60° to 60° with the scan step of 2°, 4°, and 8°. It can be seen from Figure 9 that the background uniformity of the image decreases as the number of angles reduces. Discontinuities occur on the right side of the image in Figure 9c, which appear as a sudden change in amplitude on the border where images from different angles are superimposed coherently. However, the reduction of the angle number does not affect the SNR of the defect imaging and the level of noise, which can also be seen in Figure 10. In the case of 16 plane waves, twelve defects are well presented with comparable position and amplitude to Figure 9a,b. 

Figure 10 shows the echo dynamic curves of the *x* and *z* direction under different angle numbers. It can be clearly illustrated from three amplitude distribution curves that there is a basic occurrence similarity, except for minimal discontinuities in the case of 16 plane wave angles.

**Figure 11:**
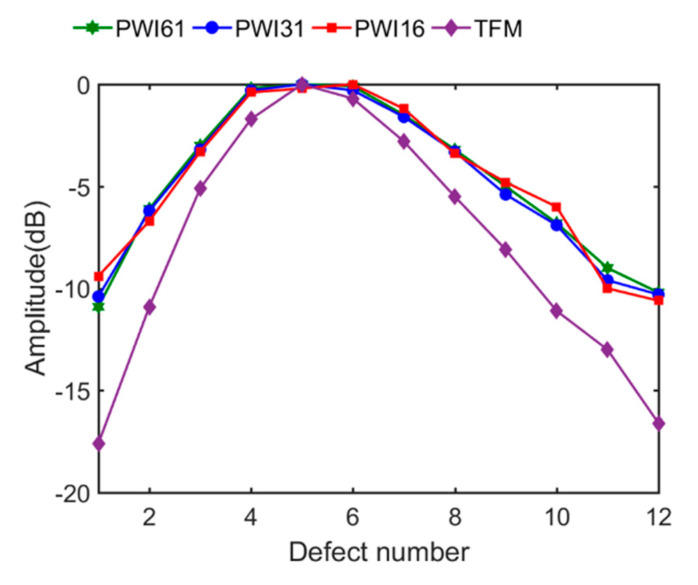
Amplitude attenuation on each defect in PWI imaging with three angle numbers and TFM imaging.

Furthermore, the defect amplitude on all 12 defects with three cases is proven in Figure 11, compared with the TFM results. In total, the amplitude attenuation of each defect remained unchanged with the number of plane waves diminished to a quarter of the original. Compared with the red curve and green curve in Figure 11, the maximum difference of amplitude was 1.5 dB on the first defect, and the average difference is only 0.48 dB. This further proves that suitable reduction of plane wave numbers does not decrease image quality. In contrast to the purple curve of TFM algorithm in Figure 11, the proposed PWI algorithm significantly improved the amplitude of all defects in general, which indicates that the spatial weighting factor optimizes the amplitude distribution in imaging regions, leading to a lower defect amplitude reduction in a large detection range, especially in the area away from the emission array.

**Figure 12:**
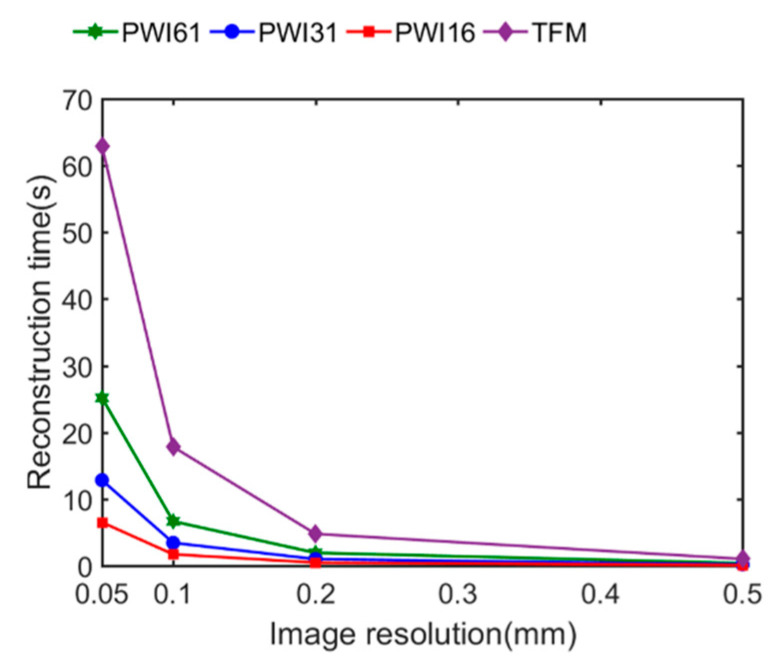
Reconstruction time in different resolutions via two algorithms.

The reconstruction times of the optimized PWI algorithm and TFM algorithm with resolutions of 0.05, 0.1, 0.2 and 0.5 mm are calculated, as plotted in Figure 12. The reconstruction time gradually decreases as the plane waves decline, and it is almost linear with the angle numbers. In particular, the 16-plane wave imaging requires only 25% of the time needed for PWI61. Additionally, the space-optimized PWI algorithm had great advantage in imaging efficiency compared with TFM. Under four conditions of different resolutions, the reconstruction time of the PWI algorithm was obviously less than that of the TFM algorithm. In the case of 0.1 mm imaging resolution, the 16-angle plane waves imaging required 1.73 s, while the TFM imaging was up to 17.83 s, i.e., reducing the reconstruction times by a factor of up to 10. Note that the number of plane waves cannot shorten indefinitely, and rare plane waves probably lead to incomplete defects imaging in the interested area. The reduction in computation time was due to the effective imaging area $E\left(x_{1},x_{n},z,\theta \right)$ defined according to the plane wave angle in the optimized plane wave algorithm, yielding a reduction of unnecessary calculations in the imaging area.

## 4. Conclusions

In this work, an optimized PWI method was proposed for the high-speed imaging of defects. The considered “gold standard” TFM was utilized to compare the imaging quality and reconstruction time, both in simulation and experiment. The testing specimen containing 12 defects was inspected by a 32-element phased array, and the defects area was subsequently imaged. The experimental and simulated results were in excellent agreement.

The space optimized PWI reached a wider detection range and lower defect amplitude attenuation without significantly increasing the amplitude of background noise, resulting in a better image quality than that of the TFM algorithm. Compared with the TFM, the proposed PWI method maximally improved 6.3 dB of defect amplitude, with a 3.1 dB improvement for an average of 12 defects. Meanwhile, less reconstruction time was obtained with a sensible reduction in the number of plane waves used in the PWI method. In the experiments, when the number of plane waves declined to a quarter, the imaging SNR and the amplitude attenuation of the defects remain at a similar level. In addition, the reconstruction time of the proposed PWI method was 10 times less than that of the TFM, which provided a promising way of defect imaging for industrial inspection. Especially when the active aperture was restricted in complex shaped specimens, dynamic detection range and imaging gain on defects away from the array were effectively promoted.

## Figures and Tables

**Table 1:** Array parameters in the simulation and experiment.

Number of Elements	Element Pitch (mm)	Active Aperture (mm)	Central Frequency (MHz)	Ultrasonic Velocity (m·s^−1^)
32	0.6	19.2	5	5930

**Table 2:** Attenuation introduced at each defect in the simulation.

Defect Number	1	2	3	4	5	6	7	8	9	10	11	12
PWI (dB)	−11.1	−5.9	−3.0	−1.0	−0.1	0	−0.9	−2.2	−3.3	−5.1	−6.5	−7.4
TFM (dB)	−16.9	−10.2	−4.5	−1.2	0	−0.3	−1.8	−4.1	−6.4	−8.7	−11.0	−13.0

**Table 3:** Attenuation introduced at each defect in the experiment.

Defect Number	1	2	3	4	5	6	7	8	9	10	11	12
PWI (dB)	−10.9	−6.1	−3.0	−0.2	0	−0.1	−1.5	−3.2	−5.0	−6.8	−9.0	−10.2
TFM (dB)	−17.6	−10.9	−5.1	−1.7	0	−0.7	−2.8	−5.5	−8.1	−11.1	−13.0	−16.6

**Table 4:** Reconstruction time by the TFM and PWI methods.

	PWI in Simulation	TFM in Simulation	PWI in Experiment	TFM in Experiment
Reconstruction time (s)	7.8	21.2	6.7	17.8

## Data Availability

Not applicable.

## References

[1] Brizuela J., Camacho J., Cosarinsky G., Iriartec J.M., Cruzab J.F. (2019). Improving Elevation Resolution in Phased-Array Inspections for NDT. NDT E Int..

[2] Zhuang Z., Zhang J., Lian G., Drinkwater B.W. (2020). Comparison of Time Domain and Frequency-Wavenumber Domain Ultrasonic Array Imaging Algorithms for Non-Destructive Evaluation. Sensors.

[3] Pérez E., Kirchhof J., Krieg F., Römer F. (2020). Subsampling Approaches for Compressed Sensing with Ultrasound Arrays in Non-Destructive Testing. Sensors.

[4] Mahaut S., Roy O., Beroni C., Rotter B. (2002). Development of Phased Array Techniques to Improve Characterization of Defect Located in a Component of Complex Geometry. Ultrasonics.

[5] Fritsch C., Parrilla M., Ibáñez A., Giacchetta R., Martínez O. (2006). The Progressive Focusing Correction Technique for Ultrasound Beamforming. IEEE Trans. Ultrason. Ferroelectr. Freq. Control.

[6] Wilcox P.D., Holmes C., Drinkwater B.W. (2007). Advanced Reflector Characterization with Ultrasonic Phased Arrays in NDE Applications. IEEE Trans. Ultrason. Ferroelectr. Freq. Control.

[7] Chiao R.Y., Thomas L.J., Silverstein S.D. (1997). Sparse Array Imaging with Spatially-Encoded Transmits. IEEE Ultrason. Symp. Proc..

[8] Holmes C., Drinkwater B.W., Wilcox P.D. (2005). Post-Processing of the Full Matrix of Ultrasonic Transmit-Receive Array Data for Non-Destructive Evaluation. NDT E Int..

[9] Yu B., Mei Y.J., Jin H.R., Wu E.Y., Yang K.J. (2018). Ultrasonic Phased Array Total Focusing Method Based on Sparse Deconvolution. J. Acoust. Soc. Am.

[10] Zhang J., Drinkwater B.W., Wilcox P.D., Hunter A.J. (2010). Defect Detection Using Ultrasonic Arrays: The Multi-Mode Total Focusing Method. NDT E Int..

[11] Camachoa J., Atehortuab D., Cruzaa J.F., Brizuelac J., Ealob J. (2018). Ultrasonic Crack Evaluation by Phase Coherence Processing and TFM and Its Aplication to Online Monitoring in Fatigue Tests. NDT E Int..

[12] Gammelmark K.L., Jensen J.A. (2003). Multielement Synthetic Transmit Aperture Imaging Using Temporal Encoding. IEEE Trans. Med. Imaging.

[13] Bannouf S., Robert S., Casula O., Prada C. (2013). Data Set Reduction for Ultrasonic TFM Imaging Using the Effective Aperture Approach and Virtual Sources. J. Phys. Conf. Ser..

[14] Martín-Arguedas C.J., Martínez-Graullera O., Romero-Laorden D., Gómez-Ullate L. (2013). Method and Architecture to Accelerate Multi-Element Synthetic Aperture Imaging. Digit. Signal Process.

[15] Moreau L., Drinkwater B.W., Wilcox P.D. (2009). Ultrasonic Imaging Algorithms with Limited Transmission Cycles for Rapid Nondestructive Evaluation. IEEE Trans. Ultrason. Ferroelectr. Freq. Control.

[16] Montaldo G., Tanter M., Bercoff J., Benech N., Fink M. (2009). Coherent Plane-Wave Compounding for Very High Frame Rate Ultrasonography and Transient Elastography. IEEE Trans. Ultrason. Ferroelectr. Freq. Control.

[17] Denarie B., Tangen T., Ekroll I., Rolim N., Torp H., Bjastad T., Løvstakken L. (2013). Coherent Plane Wave Compounding for Very High Frame Rate Ultrasonography of Rapidly Moving Targets. IEEE Trans. Med. Imaging.

[18] Cruza J.F., Camacho J., Fritsch C. (2017). Plane-Wave Phase-Coherence Imaging for NDE. NDT E Int..

[19] Merabet L., Robert S., Prada C. (2020). The Multi-Mode Plane Wave Imaging in the Fourier Domain: Theory and Applications to Fast Ultrasound Imaging of Cracks. NDT E Int..

[20] Jeune L., Robert S., Villaverde E., Prada C. (2016). Plane Wave Imaging for Ultrasonic Non-Destructive Testing: Generalization to Multimodal Imaging. Ultrasonics.

